# Supplementary material for: CARD8 and IL1B Polymorphisms Influence MRI Brain Patterns in Newborns with Hypoxic-Ischemic Encephalopathy Treated with Hypothermia
Source: Antioxidants (Basel). 2021 Jan 12;10(1):96. doi: 10.3390/antiox10010096 (PMC7826682; doi:10.3390/antiox10010096)
Supplement: Supplementary file 1 [file antioxidants-10-00096-s001.pdf]

Supplementary Materials

Table S1: Genotype frequencies of selected polymorphisms.

Gene	SNP	Role	Genotype	N (%)	MAF	pHWE
**Antioxidative pathway**						
SOD2	**rs4880**	p.Ala16Val	CC	13 (29.5)	0.420	0.271
			CT	25 (56.8)		
			TT	6 (13.6)		
CAT	**rs1001179**	c.-262C>T	CC	29 (65.9)	0.193	0.729
			CT	13 (29.5)		
			TT	2 (4.5)		
GPX1	**rs1050450**	p.Pro198Leu	CC	19 (43.2)	0.364	0.441
			CT	18 (40.9)		
			TT	7 (15.9)		
**Inflammatory pathway**						
NLRP3	**rs35829419**	p.Gln705Lys	CC	38 (86.4)	0.068	0.627
			CA	6 (13.6)		
CARD8	**rs2043211**	p.Cys10Ter	AA	21 (47.7)	0.295	0.543
			AT	20 (45.5)		
			TT	3 (6.8)		
IL1B	**rs1143623**	c.-1560G>C	GG	23 (52.3)	0.273	0.836
			GC	18 (40.9)		
			CC	3 (6.8)		
	**rs16944***	c.-598T>C	TT	5 (11.4)	0.295	0.401
			TC	16 (36.4)		
			CC	23 (52.3)		
	**rs1071676**	c.*505G>C	GG	19 (43.2)	0.352	0.721
			GC	19 (43.2)		
			CC	6 (13.6)		
TNF	**rs1800629**	c.-308 G>A	GG	31 (70.5)	0.170	0.442
			GA	11 (25.0)		
			AA	2 (4.5)		

*polymorphic C allele is more frequent in the population.

Legend: HWE= Hardy Weinberg equilibrium, MAF=minor allele frequency, SNP= single nucleotide polymorphism

Table S2: The association of polymorphisms with brainstem and cortex damage.

			**Brainstem**				**Cortex**			
**Gene**	**SNP**	**Genotype**	**Normal+ mild N (%)**	**Moderate+ severe N (%)**	**OR (95% CI)**	**P**	**Normal+ mild N (%)**	**Moderate+ severe N (%)**	**OR (95% CI)**	**P**
SOD2	rs4880	CC	9 (69.2)	4 (30.8)	Ref.		10 (76.9)	3 (23.1)	Ref.	
		CT+TT	26 (83.9)	5 (16.1)	0.43 (0.09-1.97)	0.279	24 (77.4)	7 (22.6)	0.97 (0.21-4.54)	0.971
CAT	rs1001179	CC	22 (75.9)	7 (24.1)	Ref.		21 (72.4)	8 (27.6)	Ref.	
		CT+TT	13 (86.7)	2 (13.3)	0.48 (0.09-2.69)	0.406	13 (86.7)	2 (13.3)	0.40 (0.07-2.20)	0.295
GPX1	rs1050450	CC	15 (78.9)	4 (21.1)	Ref.		15 (78.9)	4 (21.1)	Ref.	
		CT+TT	20 (80)	5 (20)	0.94 (0.21-4.1)	0.932	19 (76)	6 (24)	1.18 (0.28-4.97)	0.817
NLRP3	rs35829419	CC	29 (76.3)	9 (23.7)	Ref.		28 (73.7)	10 (26.3)	Ref.	
		CA	6 (100)	0 (0)	/	0.319*	6 (100)	0 (0)	/	0.310*
CARD8	rs2043211	AA	13 (61.9)	8 (38.1)	Ref.		13 (61.9)	8 (38.1)	Ref.	
		AT+TT	22 (95.7)	1 (4.3)	0.07 (0.01-0.66)	**0.020**	21 (91.3)	2 (8.7)	0.15 (0.03-0.84)	**0.031**
IL1B	rs1143623	GG	19 (82.6)	4 (17.4)	Ref.		19 (82.6)	4 (17.4)	Ref.	
		GC+CC	16 (76.2)	5 (23.8)	1.48 (0.34-6.48)	0.599	15 (71.4)	6 (28.6)	1.90 (0.45-7.98)	0.381
	rs16944	CC	19 (82.6)	4 (17.4)	Ref.		19 (82.6)	4 (17.4)	Ref.	
		TC+TT	16 (76.2)	5 (23.8)	1.48 (0.34-6.48)	0.599	15 (71.4)	6 (28.6)	1.90 (0.45-7.98)	0.381
	rs1071676	GG	14 (73.7)	5 (26.3)	Ref.		14 (73.7)	5 (26.3)	Ref.	
		GC+CC	21 (84)	4 (16)	0.53 (0.12-2.34)	0.405	20 (80)	5 (20)	0.70 (0.17-2.88)	0.621
TNF	rs1800629	GG	26 (83.9)	5 (16.1)	Ref.		24 (77.4)	7 (22.6)	Ref.	
		GA+AA	9 (69.2)	4 (30.8)	2.31 (0.51-10.54)	0.279	10 (76.9)	3 (23.1)	1.03 (0.22-4.80)	0.971

*calculated using Fisher's exact test

Legend: CI= confidence interval, OR=odds ratio, SNP=single nucleotide polymorphism.
